# Nanoparticle approaches for manipulating cytokine delivery and neutralization

**DOI:** 10.3389/fimmu.2025.1592795

**Published:** 2025-06-10

**Authors:** Rui Wang, Luke J. Kubiatowicz, Runhe Zhang, Lin Bao, Ronnie H. Fang, Liangfang Zhang

**Affiliations:** ^1^ Aiiso Yufeng Li Family Department of Chemical and Nano Engineering, Shu and K.C. Chien and Peter Farrell Collaboratory, University of California San Diego, San Diego, United States; ^2^ Division of Host-Microbe Systems and Therapeutics, Department of Pediatrics, University of California San Diego, San Diego, United States

**Keywords:** cytokine delivery, cytokine neutralization, nanodelivery, biodetoxification, biomimetic nanoparticle, autoimmune disease

## Abstract

Cytokines are crucial regulators of inflammation and immune tolerance, making them promising targets for treating immune-related diseases like cancer, infections, and autoimmune disorders. While cytokine-based therapies have shown potential, challenges such as dose-limiting toxicity and suboptimal pharmacokinetics have constrained their clinical success. Recent advancements in nanotechnology offer innovative solutions to these limitations, particularly through the use of nanoparticle-based platforms that enhance cytokine delivery and neutralization. This review begins by examining the landscape of cytokine delivery, emphasizing how it can be accomplished using nanoparticle systems encapsulating proteins, DNA or mRNA payloads. We then discuss recent progress on platforms for nanoparticle-based cytokine neutralization, including nanoparticle–antibody complexes and cell membrane-coated nanoparticles. Finally, we highlight the latest clinical developments in cytokine-based therapies employing these strategies before addressing the critical challenges ahead that need to be overcome in order to fully realize the therapeutic potential of nanoparticle-based cytokine manipulation.

## 1 Introduction

Cytokines are essential regulators of immune function, metabolism, and tissue development, coordinating various cellular responses to maintain health (1). In response to infections or other inflammatory signals, cytokines modulate cell-cell interactions, activating both innate and adaptive immune responses (2, 3). Primarily produced by immune cells like macrophages, lymphocytes, and T cells, cytokines are also secreted by other cell types, reflecting the complex communication between the immune systems and various tissues within the body (4–7).

Cytokines are categorized by their structure and immune function into groups, including interleukins (ILs), tumor necrosis factors (TNFs), interferons (IFNs), chemokines, and colony-stimulating factors (CSFs) (8–11), and each can have different effects on the immune system. For instance, IL-6 and IL-12 enhance cell-to-cell communication and immune activation (12–15), while IL-10 limits immune responses to protect tissues (16, 17). TNFα regulates inflammation and apoptosis (18), while IFNs play a crucial role in antiviral defenses (19, 20). Chemokines direct immune cell migration and tissue positioning, and CSFs stimulate blood cell production to meet demands during infection and recovery (10, 21, 22).

Given their central role in immune responses, cytokines play an important part in the treatment of conditions such as cancer, autoimmune diseases, and infections (23–27). Cytokine therapies typically aim to either enhance immune activity or neutralize excessive cytokine levels to manage inflammation (28–30). Approved cytokine therapies have demonstrated clinical benefits. For example, IL-2 was approved in the 1990s for treating metastatic renal cell carcinoma and melanoma due to its immune-stimulating effects (31, 32). In contrast, TNFα blockers have been essential in managing rheumatoid arthritis and Crohn’s disease by inhibiting proinflammatory cytokines, later expanding to conditions like psoriatic arthritis (33, 34).

Cytokine-based therapies can be implemented by directly delivering the protein itself, as well as by delivering the corresponding DNA or mRNA (35). Protein delivery is the most common, offering immediate effects as cytokines bind directly to cell receptors, but challenges like short half-life and potential side effects persist (36, 37). Advances in protein engineering aim to enhance stability and reduce side effects by modifying cytokine structure (38, 39). The DNA format provides a sustained therapeutic effect by encoding for cytokines that can then be expressed in cells, although delivery efficiency and safety remain concerns (40, 41). Recently, mRNA-based platforms have gained attention, as they enable quick protein synthesis in the cytoplasm without entering the nucleus (42, 43). However, challenges like mRNA stability and targeted delivery are significant hurdles (44).

In addition to cytokine delivery, regulating cytokine overproduction is crucial for managing conditions such as autoimmune diseases, chronic inflammation, and allergic reactions (26, 29, 45, 46). Excessive levels of proinflammatory cytokines such as TNFα drive persistent inflammation, resulting in tissue damage (3, 47). In severe cases, cytokine storms—characterized by the significant overproduction of multiple proinflammatory cytokines—have contributed to the mortality observed in patients suffering from severe infections (48). To mitigate elevated cytokine levels, neutralization strategies have been developed. Monoclonal antibodies are widely used in this context, as they can specifically bind to cytokines or their receptors, blocking interactions with immune cells and effectively reducing excessive inflammatory responses (49, 50). Additionally, small molecule inhibitors can be used to target key signaling pathways like JAK-STAT, which many inflammatory cytokines utilize (51, 52). These inhibitors are effective in treating conditions such as rheumatoid arthritis and ulcerative colitis (53, 54). Despite the efficacy of these immunosuppressive agents, challenges remain, particularly in achieving specificity, minimizing off-target effects, and ensuring controlled delivery (55, 56).

Systemic cytokine administration often faces limitations such as rapid degradation and nonspecific interactions, impacting clinical efficacy (23, 57). Nanoparticles offer solutions by enabling controlled release, protection from degradation, and targeted delivery (58, 59). With features like high surface area-to-volume ratios, tunable sizes, and customizable surfaces, nanoparticles can improve the efficacy and safety of cytokine therapy (60, 61). Nanoparticles can deliver cytokines in protein, DNA, or mRNA formats tailored to specific therapeutic needs (36, 62). For protein delivery, nanoparticles can release cytokines at the desired target site to ensure precise receptor interactions, while for DNA or mRNA delivery, they are able to protect the nucleic acids from enzymatic degradation, enhancing cellular uptake and endosomal escape (35, 42, 63). On the other side of things, nanoparticle-based cytokine neutralization platforms, including nanoparticle–antibody complexes and cell membrane-coated nanoparticles (CNPs), show substantial potential in addressing the limitations of traditional antibody therapies (64).

**Figure 1:**
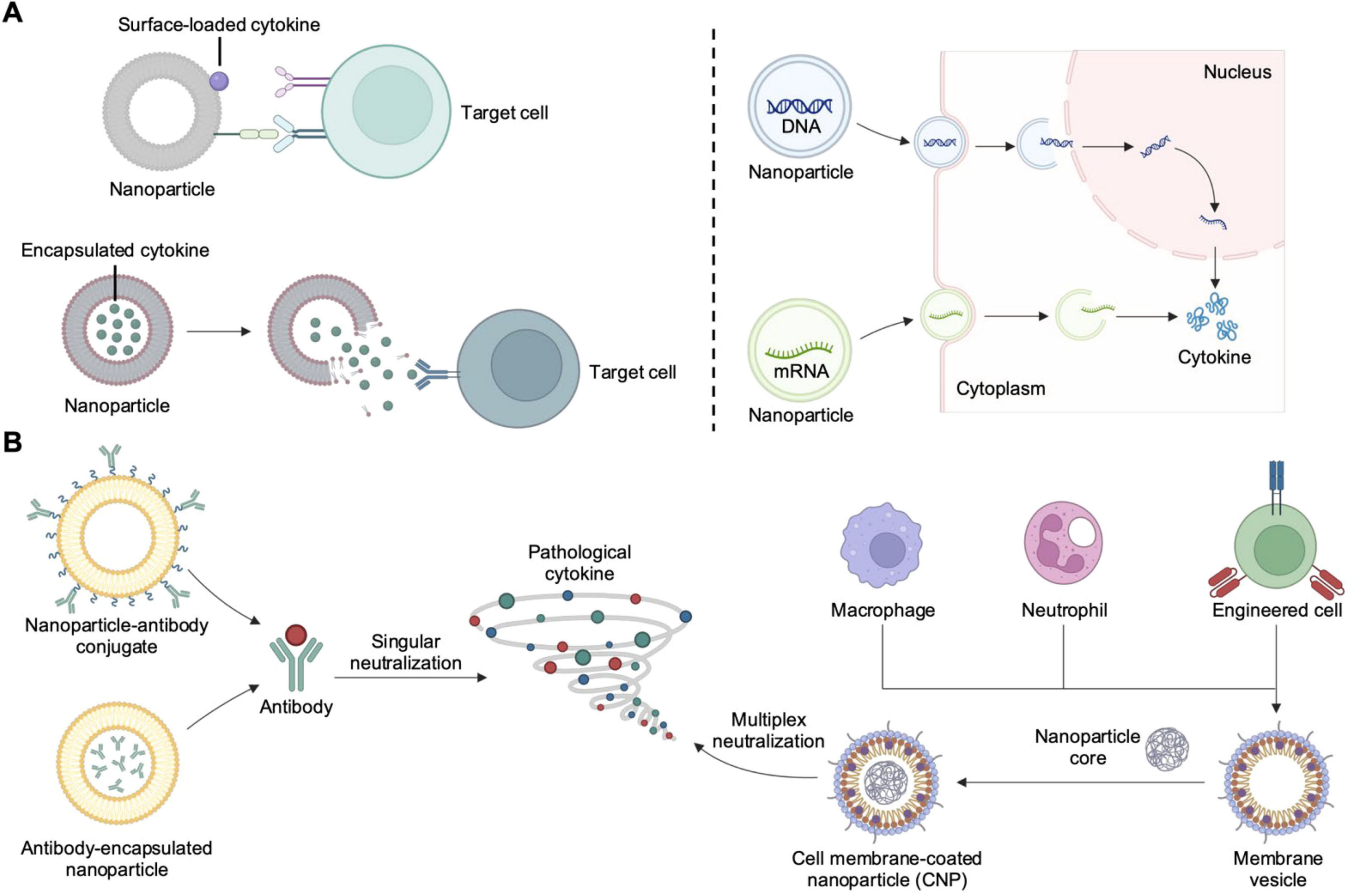
Nanoparticle-based strategies for cytokine delivery and neutralization. **(A)** Nanoparticles can deliver cytokines in the form of protein, DNA, or mRNA. Proteins can be encapsulated for environment-responsive release or surface-immobilized for improved receptor engagement. DNA or mRNA can be protected from degradation while experiencing enhanced localization to the appropriate subcellular compartment. **(B)** Nanoparticle-based cytokine neutralization strategies include the used of nanoparticle–antibody complexes and cell membrane-coated nanoparticles (CNPs). Created with BioRender.

This review comprehensively examines recent advances in nanoparticle-based approaches for cytokine delivery and neutralization (**Figure 1**). We begin by detailing innovative nanoparticle-based strategies for delivering cytokines in the form of protein, DNA, or mRNA. Next, we explore methods for nanoparticle-based cytokine neutralization, emphasizing nanoparticle–antibody complexes and CNPs. Finally, we summarize the current state of clinical translation for cytokine therapies, looking ahead to the key challenges that must be tackled for nanoparticle-based platforms to achieve their full therapeutic potential.

## 2 Nanoparticle-based cytokine delivery

### 2.1 Protein delivery

#### 2.1.1 Cytokine protein engineering

**Figure 2:**
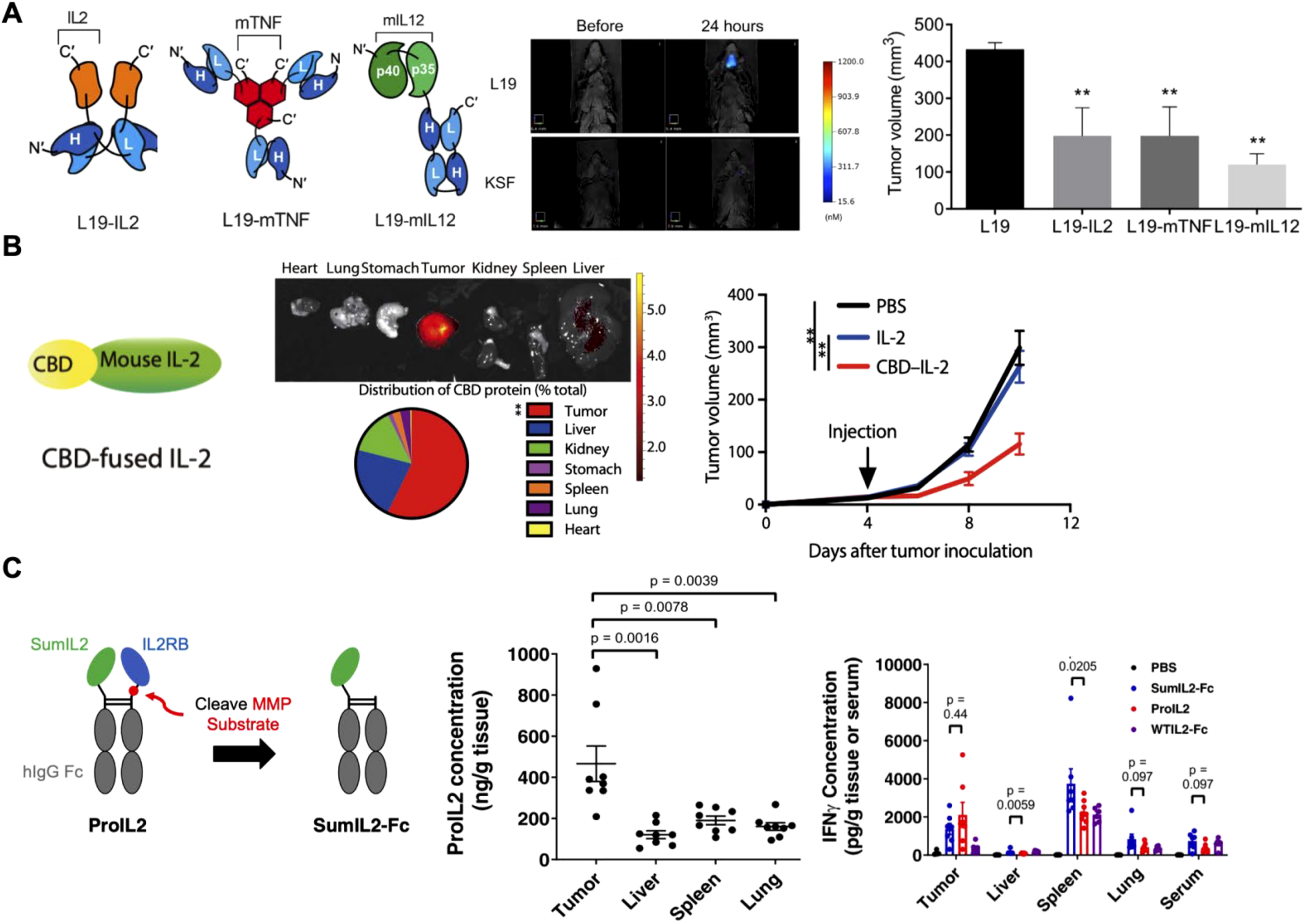
Cytokine fusion constructs for improved delivery. **(A)** Immunocytokines formed by fusing the L19 antibody with cytokines such as IL-2, TNF, and IL-12 show selective accumulation in tumor tissues, thus enhancing antitumor immune responses. Adapted with permission (69). Copyright 2020, American Association for the Advancement of Science. **(B)** IL-2 fused to the von Willebrand factor A3 domain, a collagen-binding domain (CBD), exhibits improved safety and efficacy after delivery by systemic administration. Adapted with permission (73). Copyright 2019, American Association for the Advancement of Science. **(C)** IL-2 linked to its receptor via a protease-sensitive linker activates only in the tumor microenvironment, enhancing antitumor immune responses. Adapted with permission (75). Copyright 2021, Nature Publishing Group. **p < 0.01.

Engineering cytokines by fusing them with functional proteins can enhance their therapeutic potential by improving target specificity and reducing challenges like poor pharmacokinetics and high off-target toxicity (35). Immunocytokine therapy combines cytokines with targeting modules such as antibodies, which direct the cytokines to specific cells or tissues (65). For instance, cytokines fused with antibodies against tumor markers like CD20 (66), programmed death protein 1 (67), and extra-domain B (EDB) of fibronectin (68) have shown promising results in cancer therapies and are currently being tested in clinical trials. One notable example is the fusion of inflammatory cytokines with the L19 antibody, which selectively targets the EDB of fibronectin (**Figure 2A**) (69). This marker is highly expressed in glioblastoma tissue but absent in healthy adult tissues, making it an ideal target for cancer treatment. Through genetic engineering, researchers have developed various L19-based immunocytokines to deliver cytokines such as IL-2, TNF, and IL-12 and studied their biodistribution in tumor-bearing mice after systemic administration. These immunocytokines preferentially accumulated in tumor tissue and its blood vessels, avoiding healthy brain tissue. Intravenous injection of the fusion proteins resulted in partial tumor regression, while further studies demonstrated increased lymphocyte infiltration and the release of other proinflammatory cytokines within the tumor microenvironment, thereby strengthening the antitumor immune response.

Besides antibody conjugation, fusing cytokines to other functional ligands with inherent targeting properties offers another approach to enhance cytokine specificity (70). In the context of cancer therapies, collagen—a predominant component in the tumor microenvironment (TME)—serves as an effective anchor. Several collagen-binding domains (CBDs) from functional proteins have been fused with proinflammatory cytokines, such as IL-2 or IL-12, to guide them directly to collagen within the TME (71, 72). For instance, the A3 CBD domain of von Willebrand factor (VWF) was fused with IL-2 to facilitate targeted cytokine delivery to tumors, capitalizing on the leaky vasculature typical of tumor tissues (**Figure 2B**) (73). *In vivo* biodistribution studies demonstrated that after a single intravenous injection, the CBD-fused cytokine selectively accumulated in tumor sites, with lower accumulation in organs like the kidney and liver, which have fenestrated endothelia. Additionally, compared to free IL-2, the CBD-IL-2 fusion protein showed enhanced antitumor effects across various cancer models and significantly reduced systemic toxicity.

To mitigate off-target effects, researchers have developed prodrug systems where cytokines are linked to their receptors by cleavable linkers, enabling the cytokine to be activated only upon reaching the target site (74). One example involved fusing IL-2 to its receptor via a tumor protease-sensitive linker (**Figure 2C**) (75). In this case, IL-2 receptor β (IL2Rβ) was used to bind and inhibit IL-2 activity prior to cleavage, which converted it into its active form following matrix metalloproteinase-mediated cleavage within tumor tissues. Biodistribution studies revealed that the prodrug accumulated in tumors at 2 to 4 times the levels observed in other tissues. Furthermore, in comparison to using the active form directly, the IL-2 prodrug elicited similar cytokine production within tumor sites while reducing peripheral inflammatory cytokine levels. This effectively minimized toxicity and mortality while preserving antitumor effectiveness.

#### 2.1.2 Surface-anchored cytokine protein delivery

While fusing cytokines with functional moieties or converting them into prodrugs enhances targeting and bioavailability, the clinical outcomes may remain limited by factors such as the need for local administration, suboptimal pharmacokinetics, and monofunctionality (76). The integration of cytokines with nanoparticles has emerged as a promising strategy to overcome these limitations by improving bioavailability and enhancing biodistribution, thereby directing cytokines to specific organs or immune cells of interest (58). Nanoparticles offer a versatile platform that combines the benefits of traditional fusion proteins with improved stability, controlled release, multifunctionality, and reduced toxicity (77, 78). This adaptability opens new possibilities for more effective cytokine therapies (63).

**Figure 3:**
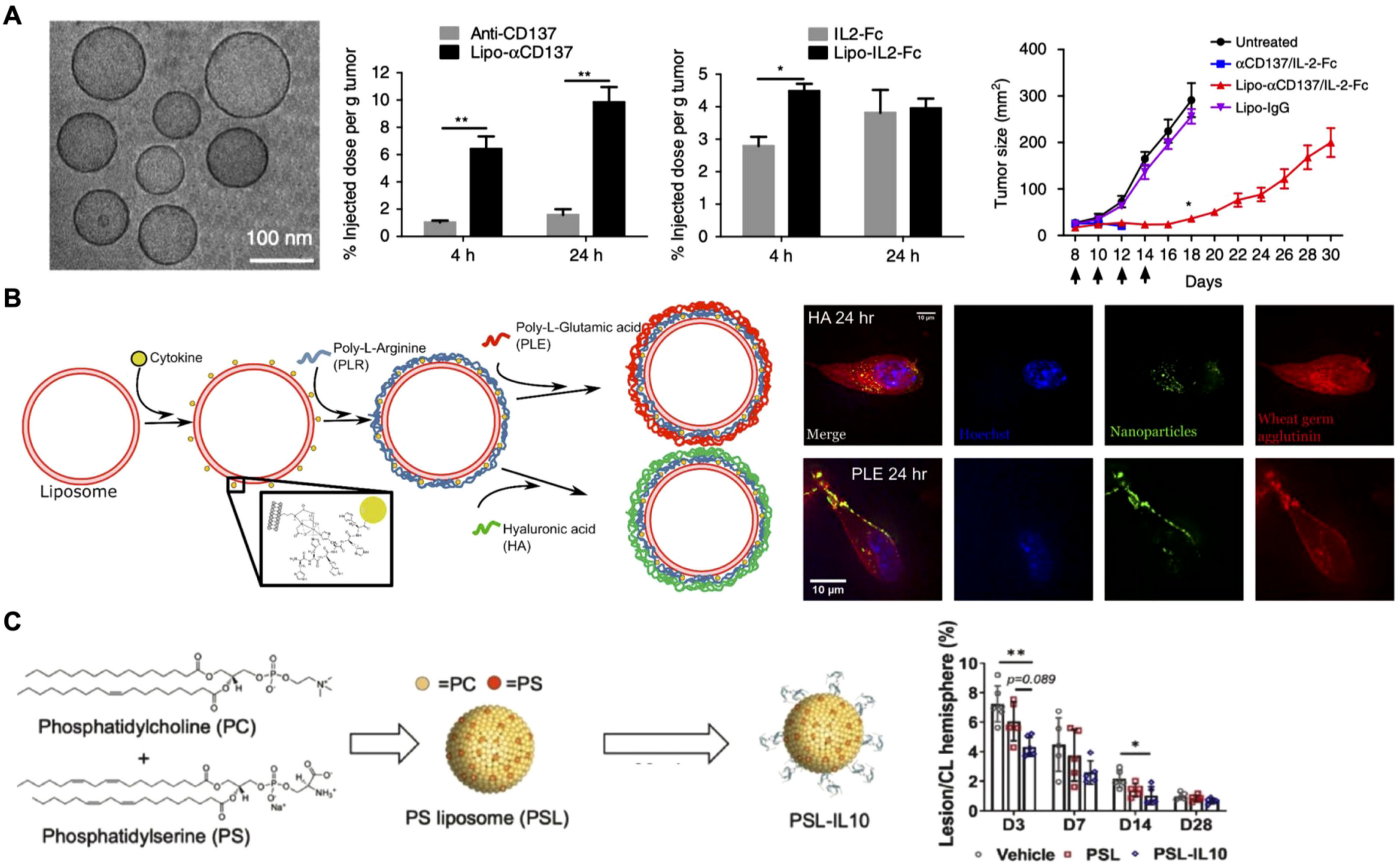
Surface-anchored cytokines on nanoparticles. **(A)** IL-2 and anti-CD137 are anchored on stealth liposomes, enabling them to achieve rapid tumor accumulation and enhanced antitumor effects. Adapted with permission (83). Copyright 2018, Nature Publishing Group. **(B)** IL-12-loaded liposomes are coated with hyaluronic acid (HA) and poly-L-glutamic (PLE) acid through layer-by-layer assembly, and the resulting formulation effectively targets cancer and immune cells. HA-coated liposomes show efficient uptake by cancer cells, while PLE-coated liposomes demonstrate prolonged IL-12 retention at the tumor site. Adapted with permission (85). Copyright 2020, American Chemical Society. **(C)** Phosphatidylserine liposomes functionalized with IL-10 selectively target microglia and macrophages, enhancing recovery and reducing inflammation after intracerebral hemorrhage. Adapted with permission (87). Copyright 2023, Elsevier. *p < 0.05 and **p < 0.01.

Surface anchoring of cytokines on nanoparticles facilitates direct receptor interaction and enhances therapeutic effectiveness (35). Researchers have explored several methods to improve the localization and prolong the retention of cytokines at target sites (79–81). In tumor therapy, the enhanced permeability and retention (EPR) effect allows nanoparticles to passively accumulate more efficiently in tumor tissues, making them ideal carriers for delivering proinflammatory cytokines such as IL-2, IL-12, and TNFα (82). This strategy was taken one step further by anchoring IL-2 along with the immunostimulatory agonist anti-CD137 on the surface of stealth liposomes modified with polyethylene glycol (PEG) to improve tumor accumulation by active targeting (**Figure 3A**) (83). Earlier studies had revealed that a fusion protein consisting of IL-2 and anti-CD137 produced strong antitumor effects but was still accompanied by severe systemic toxicity (84) Thus, researchers designed the liposome-based formulation aimed at achieving rapid tumor localization with minimal systemic exposure during *in vivo* tests. Pharmacokinetic and biodistribution studies confirmed liposome-anchored IL-2 showed faster clearance from the bloodstream but greater retention within the tumor. Antitumor efficacy evaluations demonstrated that these immunoliposomes maintained the potent therapeutic effect of IL-2 while reducing systemic toxicity across multiple tumor models.

In addition to leveraging the EPR effect of nanoparticles, targeting ligands can be anchored on the nanoparticle surface to actively direct cytokines to specific cells or tissues. One innovative strategy employed the layer-by-layer assembly technique to coat liposomes loaded with IL-12 (**Figure 3B**) (85). The outer layer was composed of either hyaluronic acid (HA) or poly-L-glutamic acid (PLE). HA was selected for its well-documented interaction with CD44, a receptor overexpressed in various cancers, including triple-negative breast cancer, non-small cell lung cancer, and ovarian cancer. On the other hand, PLE was chosen for its strong association with ovarian cancer cells and immune cells. *In vitro* fluorescence microscopy revealed distinct behaviors: liposomes with HA as the terminal layer were effectively internalized by two cancer cell lines, whereas PLE-coated liposomes bound to the cell surface membrane without significant uptake across multiple cell types. Ultimately, PLE was selected for the final nanoparticle formulation, as it ensured prolonged IL-12 interaction within the tumor microenvironment. *In vivo* studies further confirmed that PLE-coated IL-12-loaded nanoparticles effectively slowed tumor progression across several tumor models when used as monotherapy. Importantly, the nanoparticles also reduced the systemic toxicity of IL-12 across multiple dosing regimens, offering a promising strategy for safer and more targeted cytokine delivery.

In addition to proinflammatory cytokines for cancer therapy, anti-inflammatory cytokines like IL-10 have also been incorporated onto nanoparticles to prevent excessive immune activation and to protect from tissue damage (86). For example, IL-10 was conjugated onto phosphatidylserine liposomes for targeted delivery to microglia and macrophages in intracerebral hemorrhage, a severe form of stroke (**Figure 3C**) (87). *In vitro* and ex vivo studies showed that liposome-bound IL-10 was preferentially taken up by cells within the brain. Therapeutic assessments revealed significant improvements in recovery, accelerated hematoma clearance, and reduced inflammation, highlighting the potential of IL-10-loaded nanoparticles for the targeted treatment of intracerebral hemorrhage.

#### 2.1.3 Encapsulated cytokine protein delivery via nanoparticles

**Figure 4:**
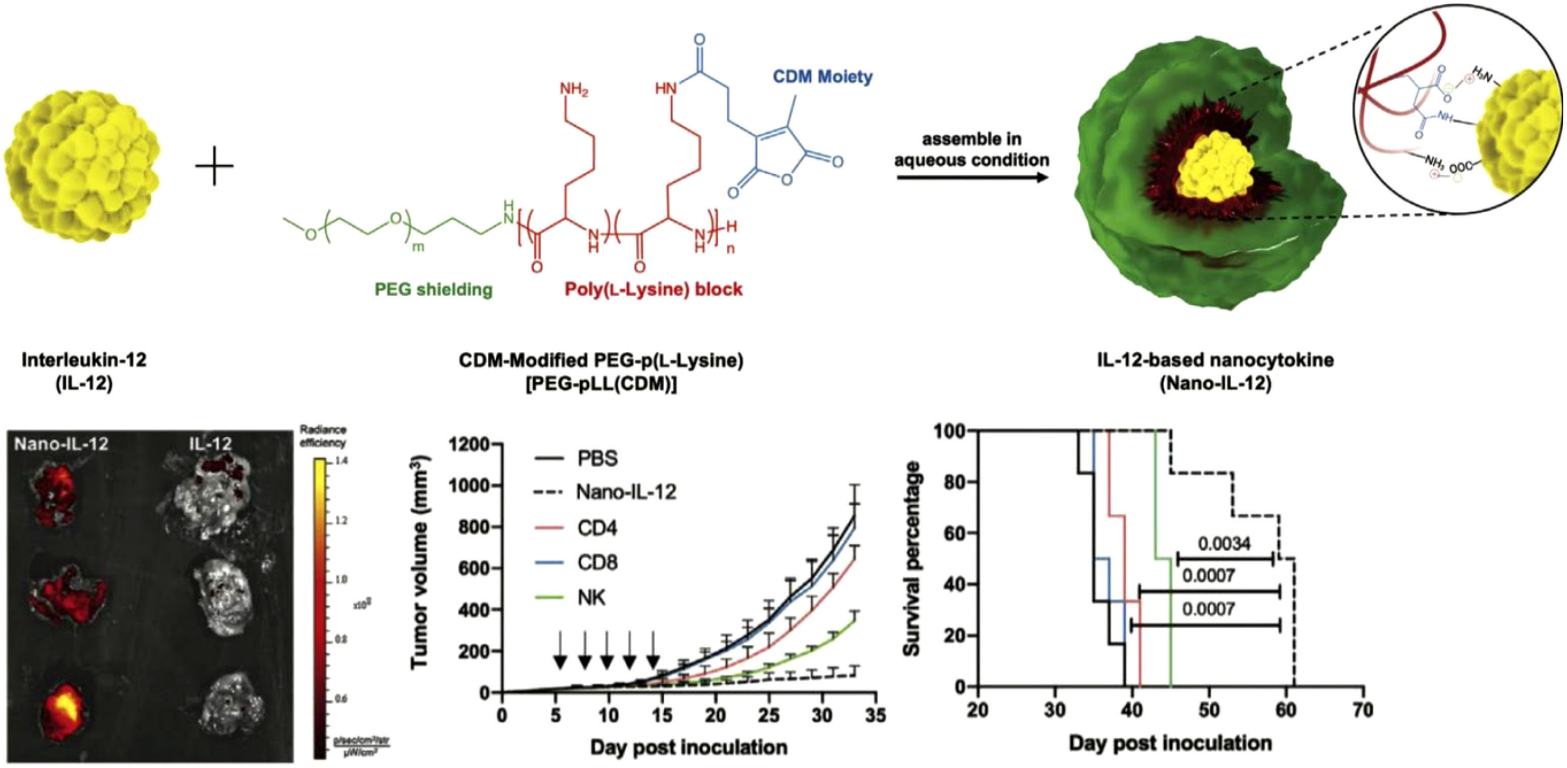
Cytokines encapsulated within nanoparticles. An IL-12 nanocytokine is constructed through the use of pH-sensitive amide bonds and electrostatic interactions, exhibiting higher tumor accumulation and enhanced antitumor effects compared to free IL-12. Adapted with permission (91). Copyright 2023, Wiley-VCH.

Encapsulating cytokines within environment-responsive nanoparticles enables targeted release in response to specific triggers, such as pH, temperature, or enzymatic activity (88). This approach addresses challenges like rapid degradation and systemic toxicity by releasing cytokines only at the desired site (89, 90). For example, IL-12 encapsulated in pH-sensitive polymers composed of modified PEG-poly(L-lysine) was designed to be released specifically in the acidic environment of the tumor microenvironment (**Figure 4**) (91). Blood circulation profiles and biodistribution studies showed that the encapsulated IL-12 remained stable during systemic circulation, achieving higher accumulation in tumors compared to free IL-12. Moreover, treatment with the nanoformulation resulted in significant antitumor effects, which was attributed to increased infiltration of immune cells into the tumor microenvironment that enhanced the overall therapeutic impact.

### 2.2 Nucleic acid delivery

#### 2.2.1 DNA delivery

**Figure 5:**
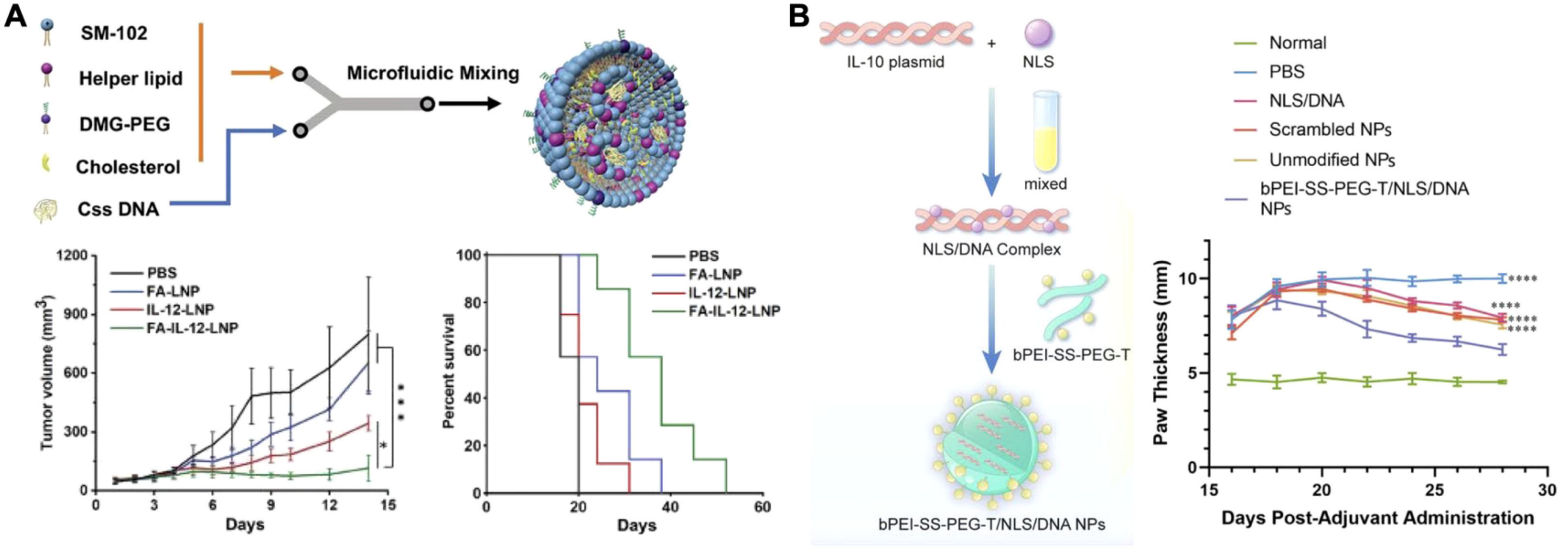
Nanoparticle-based cytokine DNA delivery. **(A)** IL-12 circular single-stranded DNA encapsulated into a folate-targeted lipid nanoparticle can be delivered via intravenous injection for antitumor therapy. Adapted with permission (101). Copyright 2024, Wiley-VCH. **(B)** An IL-10 plasmid DNA complexed with a nuclear localization signal and encapsulated within a polymeric nanoparticle can be applied for the treatment of rheumatoid arthritis. Adapted with permission (109). Copyright 2024, Elsevier. *p < 0.05 and ****p < 0.0001.

The delivery of cytokine-encoding DNA using nonviral vectors has demonstrated potential (92). As the DNA, usually in plasmid form, localizes to a target cell’s nucleus, stable and long-lasting expression of the cytokine at the site of action can be achieved (93). Due to the advantages of prolonged expression of proinflammatory signals in the local TME for cancer treatment, many research groups have sought to utilize DNA-based cytokine production as an immunotherapy. Cytokines such as LIGHT (TNF superfamily member 14) (94), IL-21 (95), and IL-12 (96–100) are good examples that have been delivered via DNA constructs for this purpose. One novel nanomedicine utilized a form of circular single-stranded IL-12 DNA delivered by lipid nanoparticles (LNPs) for targeted tumor therapy (**Figure 5A**) (101). This form of DNA was more difficult for the innate immune system to detect, but still enabled the long-term protein production of IL-12. The DNA was formulated into LNPs with various helper lipids via microfluidic mixing. For *in vivo* experiments using a 4T1 tumor model, a folate ligand was added to the exterior to enhance tumor targeting following intravenous injection. The folate-functionalized LNP formulation showed significant inhibition of tumor growth and extended survival time compared to untargeted LNPs and other controls. Follow-up studies showed that this treatment strategy significantly increased the number of CD4^+^ and CD8^+^ T cells in the tumor, as well as significantly increased IL-12 concentrations 11 days post treatment compared to a PBS control. Moreover, it was demonstrated that the targeted LNPs also significantly decreased the number of metastases.

Research groups are also seeking to utilize proangiogenic and anti-inflammatory cytokines for therapeutic purposes. These include vascular endothelial growth factor (VEGF) for ischemic heart disease (102), IL-4 for myocardial infarction (103), and IL-10 to treat a host of inflammatory conditions (104–108). In one such case, a macrophage-hitchhiking strategy was paired with an IL-10 plasmid for the treatment of rheumatoid arthritis (**Figure 5B**) (109). The plasmid was complexed with nuclear localization signal peptides to enhance DNA trafficking to the site of transcription. The complex was subsequently incubated with a previously synthesized tuftsin-modified, disulfide-crosslinked polyethylenimine (PEI)/PEG copolymer to form positively charged nanoparticles. The polymer was chosen for its ability to facilitate macrophage uptake via phagocytosis, endosomal escape via the proton sponge effect, and enhanced cytoplasmic release by glutathione cleavage. The nanoparticle reprogramming capacity was assessed with lipopolysaccharide-primed J774A.1 macrophages, where treatment resulted in increased IL-10 secretion, decreased iNOS mRNA, and increased Arg-1 and CD206 mRNA expression. This indicated a shift towards the M2 macrophage phenotype. In a collagen-induced arthritis model, the nanoparticles were administered via intraperitoneal injection. Transfected macrophages subsequently trafficked to the arthritic site, whereby IL-10 secretion helped to downregulate local inflammation. Two weeks following a single injection of the formulation, treated rats showed significantly reduced paw thickness and ankle width, along with a significant improvement in their local cytokine profiles compared to controls.

Researchers have also prioritized the optimization nanocarrier composition to improve delivery efficiency and therapeutic outcomes. For example, a custom-designed nanocarrier for IL-10 DNA delivery was developed by screening a library of cationic polymers using high-throughput testing in macrophages (110). The optimal formulation, characterized by the highest IL-10 protein expression and macrophage polarization, demonstrated preferential distribution to injured muscle, enabling sustained IL-10 production following both intramuscular and intravenous injection.

#### 2.2.2 mRNA delivery

RNA-based medicine has emerged as an alternative to DNA-based medicine due to technological breakthroughs such as the Nobel Prize winning development of nucleoside base modifications (111), which alongside other design features have endowed synthetic RNA constructs with greater stability and decreased immunogenicity (112). RNAs are advantageous over DNAs in that they can be directly translated in the cytoplasm of their target cell and come with significantly decreased risk of genomic integration. Although translation-capable RNAs are being developed in several formats like saRNA and circRNA, the most widely studied is mRNA (113). Protein production by mRNA constructs does not occur for as long as their DNA counterparts, but this does offer greater control over patient dosing. In most cases, this is a distinct advantage for mRNA-mediated cytokine delivery, given the careful balance that must be struck between safety and potency. Like DNA-based cytokine therapies, many preclinical research efforts have focused on IL-12 for tumor treatment.

Given the inherent risk of systemic IL-12 administration, it is important that exogenous use of this cytokine be restricted to the local TME. This problem was addressed by one research group through the delivery of IL-12 mRNA to lung tumors using inhalable, mRNA-loaded exosomes (114). The sequence for the IL-12 mRNA encoded for both of its subunits joined by a linker to enable cytokine production via a single transcript. Following synthesis, the mRNA was loaded via electroporation into either human embryonic kidney cell exosomes or control synthetic liposomes. These sub-200 nm formulations were subsequently nebulized into a chamber for delivery to mice by inhalation. Biodistribution analysis of dye-tagged complexes revealed the advantage of exosomes in terms of both total delivery to the lung and specificity, as liposomes showed nonspecific signal in the liver and kidneys, which could lead to off-target effects. It was further confirmed that IL-12 protein production occurred in the lungs of tumor-bearing mice after administration. This also led to increased percentages of CD8^+^ and CD4^+^ T cells in the lungs 3 days post treatment. Efficacy studies were then conducted using LL/2 and B16F10 orthotopic lung tumor models. In both cases, exosomes delivering IL-12 mRNA significantly delayed tumor growth and improved overall survival compared to PBS and the liposomal control. Regarding the mechanism of action, it was determined that the induction of IFNγ, CD8^+^ T cells, and conventional type 1 dendritic cells played the largest roles. Similar efficacy results and mechanistic findings were observed by another group that delivered IL-12 mRNA to tumors via intratumoral injection of LNPs (115). Interestingly, the mRNA used in this study incorporated an miR-122-binding site in its 3’UTR. This was used to inhibit off-target IL-12 production in the liver through mRNA degradation without significantly reducing production in the tumor, adding a safety factor. This study also employed an ex vivo human patient tumor slice culture model to investigate MEDI1191, a human variant of the mouse IL-12 mRNA therapy used throughout the study. Their findings showed dose-dependent increases in hIL12p70 production as well as subsequent induction of IFNγ and CXCL10.

**Figure 6:**
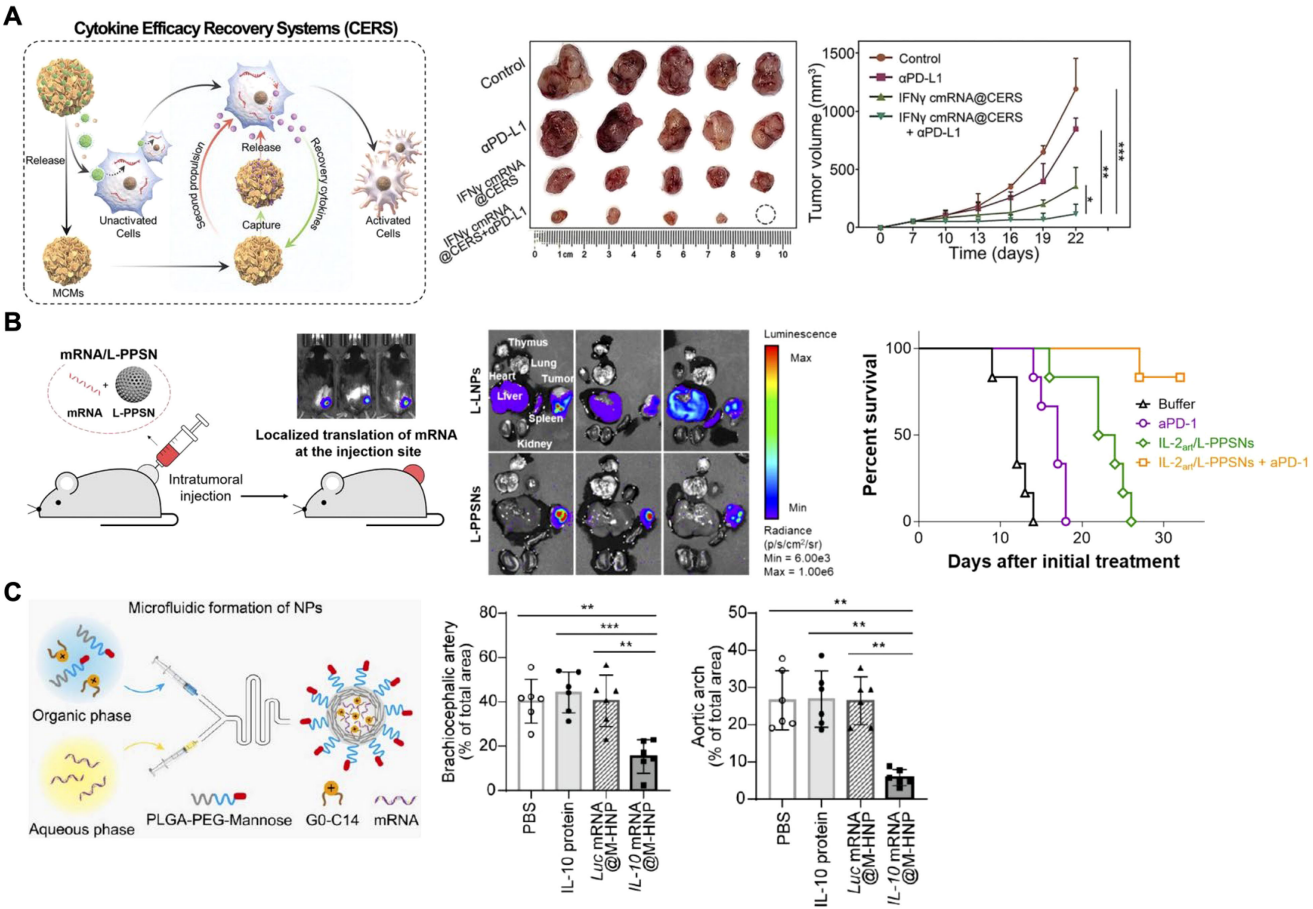
Nanoparticle-based cytokine mRNA delivery. **(A)** Nanoparticulate IFNγ mRNA delivery can be paired with a mineralized microparticle for synergistic activation of tumor-associated macrophages. Adapted with permission (120). Copyright 2023, Wiley-VCH. **(B)** IL-2 mRNA delivered via large porous silica nanoparticles by intratumoral injection mediates local cytokine production. Adapted with permission (121). Copyright 2023, American Chemical Society. **(C)** IL-10 mRNA delivered to atherosclerotic plaques via a mannose-targeted polymeric nanoparticle reduces inflammation and plaque size. Adapted with permission (129). Copyright 2023, American Chemical Society. *p < 0.05, **p < 0.01 and ***p < 0.001.

While IL-12 has been highly studied for tumor therapy (116–119), there are many other mRNA-based proinflammatory cytokine therapies that are also under investigation involving the use of IFNγ (120), IL-2 (121), and IL-15 (122). For example, IFNγ mRNA delivered by galactose-modified LNPs attached to mineral-coated microparticles enabled researchers to engineer *in vivo* cytokine efficacy recovery systems, an idea that was based on the principles of kinetic energy recovery systems used by hybrid motor vehicles (**Figure 6A**) (120). The concept works by releasing LNPs from the microparticle complex to deliver IFNγ mRNA to tumor-associated macrophages (TAMs) following intratumoral injection. This induces the secretion of IFNγ to the TME for autocrine activation. The mineral-coated microparticles are then capable of capturing secreted IFNγ via electrostatic interactions to prevent their escape from the TME. When eventually released through microparticle degradation, these captured IFNγ cytokines participate in a secondary activation of TAMs to ramp up macrophage polarization and antitumor activity. When paired with anti-programmed death-ligand 1 therapy, this approach significantly inhibited the growth of K7M2 osteosarcoma tumors in murine models. In another example of mRNA-based proinflammatory cytokine therapy, engineered IL-2 mRNA was loaded into polyethyleneimine-modified porous silica nanoparticles and delivered via intratumoral injection (**Figure 6B**) (121). The nanoparticles used in this study were designed to be large and rigid (~340 nm in diameter with ~6 nm pore size), which was hypothesized to increase their retention within the TME by preventing leakage. When compared to large and malleable LNPs ~280 nm in diameter in an intratumoral injection of firefly luciferase mRNA, the new nanoplatform showed an ~100-fold advantage in tumor/abdomen signal ratio by the second day post injection. This was subsequently found to result in significant improvements to survival in a B16F10 tumor model. In addition, an engineered IL-2 sequence was also successfully investigated and utilized in this study. The altered IL-2 cytokine reduced binding to IL-2Rα for decreased adverse effects while improving antitumor efficacy compared to the wild-type IL-2 cytokine. Efficacy was further augmented when the engineered IL-2 mRNA therapy was combined with anti-programmed death protein 1 therapy.

As is the case with DNA-based cytokine treatments, researchers have found success using mRNA constructs of angiogenic and anti-inflammatory cytokines to develop treatments for diseases other than cancer. This has included VEGF-A for ischemic tissues in cardiovascular disease (123), bone morphogenetic protein-2 and transforming growth factor beta-3 for craniofacial defect healing (124), IL-1Rα for temporomandibular joint osteoarthritis (125), IL-10 for inflammation (126, 127) and even oral delivery of IL-22 for ulcerative colitis (128). An increasingly important example of this can be seen in the application of IL-10 mRNA delivered by a nanoparticle targeting atherosclerosis (**Figure 6C**) (129). The researchers utilized positively charged G0-C14 to condense IL-10 mRNA within a poly(lactic-*co*-glycolic acid)-PEG-mannose nanoparticle. Mannose was chosen as a targeting ligand to facilitate M2-type macrophage uptake and mRNA delivery within atherosclerotic plaques. The polymeric nanoparticles were shown to effectively encapsulate mRNA, protecting it from degradation by RNase exposure. When used to treat to bone marrow-derived macrophages, the formulation showed significantly improved mRNA delivery, IL-10 production, and anti-inflammatory cytokine profile. Further *in vitro* studies showed that IL-10 mRNA delivery via the nanoparticles significantly reduced macrophage apoptosis during exposure to hydrogen peroxide and increased expression of anti-apoptotic mRNAs. The authors then moved to *in vivo* studies in which Ldlr^-/-^ mice fed on a western diet were intravenously administered with the nanoformulation. It was demonstrated that the nanoparticles were able to better target the aorta of these mice compared with healthy mice, thus confirming that the platform exhibited a level of specificity for atherosclerotic plaques. In a therapeutic efficacy study, delivery of IL-10 mRNA using the nanoparticles resulted in significantly reduced plaque area in the brachiocephalic artery, aortic arch, and aortic root compared to both an untargeted formulation and free IL-10 protein controls. A more detailed analysis of the aortic root further revealed the presence of a significantly more anti-inflammatory cytokine profile compared to the other controls.

## 3 Nanoparticle-based cytokine neutralization

Cytokine neutralization has become a critical area of research for treating inflammatory conditions like sepsis, COVID-19, and autoimmune disorders like arthritis (130–132). Currently, free neutralizing antibodies are widely used to manage these conditions characterized by pathological cytokine production (29, 133). For example, IL-6 levels increase significantly during neuroinflammation, and anti-IL-6 antibodies are commonly employed to inhibit this response (12, 13). Medications like secukinumab and clazakizumab, which use monoclonal antibodies targeting IL-6, have shown potential in treating rheumatoid arthritis, although secukinumab’s development was discontinued due to regulatory concerns, which is attributed to the fact that free antibodies often face limitations such as off-target toxicity (134–136). Without a specific targeting mechanism, they circulate systemically and can bind to healthy tissue, causing unintended side effects (137).

To address these issues, researchers have explored combining antibodies with biomaterials, which can improve their pharmacokinetics and biodistribution, resulting in a more favorable therapeutic profile (64). Various biomaterial platforms, including different scaffolds and nanoparticles, have been used explored (138). For instance, antibodies targeting IL-1β and TNFα have been conjugated to high molecular-weight polysaccharides, localizing antibody activity and minimizing systemic side effects (139). Another approach involves the use of scaffold conjugates designed to control local inflammation (140, 141). In a burn model, researchers modified hyaluronic acid with active ester groups, allowing it to be functionalized with anti-TNFα antibodies (142). This resulting platform reduced burn progression and necrosis by approximately 70% and limited macrophage infiltration, again demonstrating the effectiveness of localized cytokine neutralization.

### 3.1 Nanoparticle–antibody complexation

To further enhance the specificity and durability of cytokine-neutralizing therapies, recent advancements have focused on integrating antibodies with nanoparticle systems (64, 138). These nanoparticle–antibody complexes offer significant improvements over free antibodies, allowing for more controlled biodistribution and retention at inflammation sites (143, 144). By either attaching antibodies to the nanoparticle surface or encapsulating them within the nanoparticle structure, these approaches can enable targeted cytokine neutralization.

#### 3.1.1 Nanoparticle surface modification

**Figure 7:**
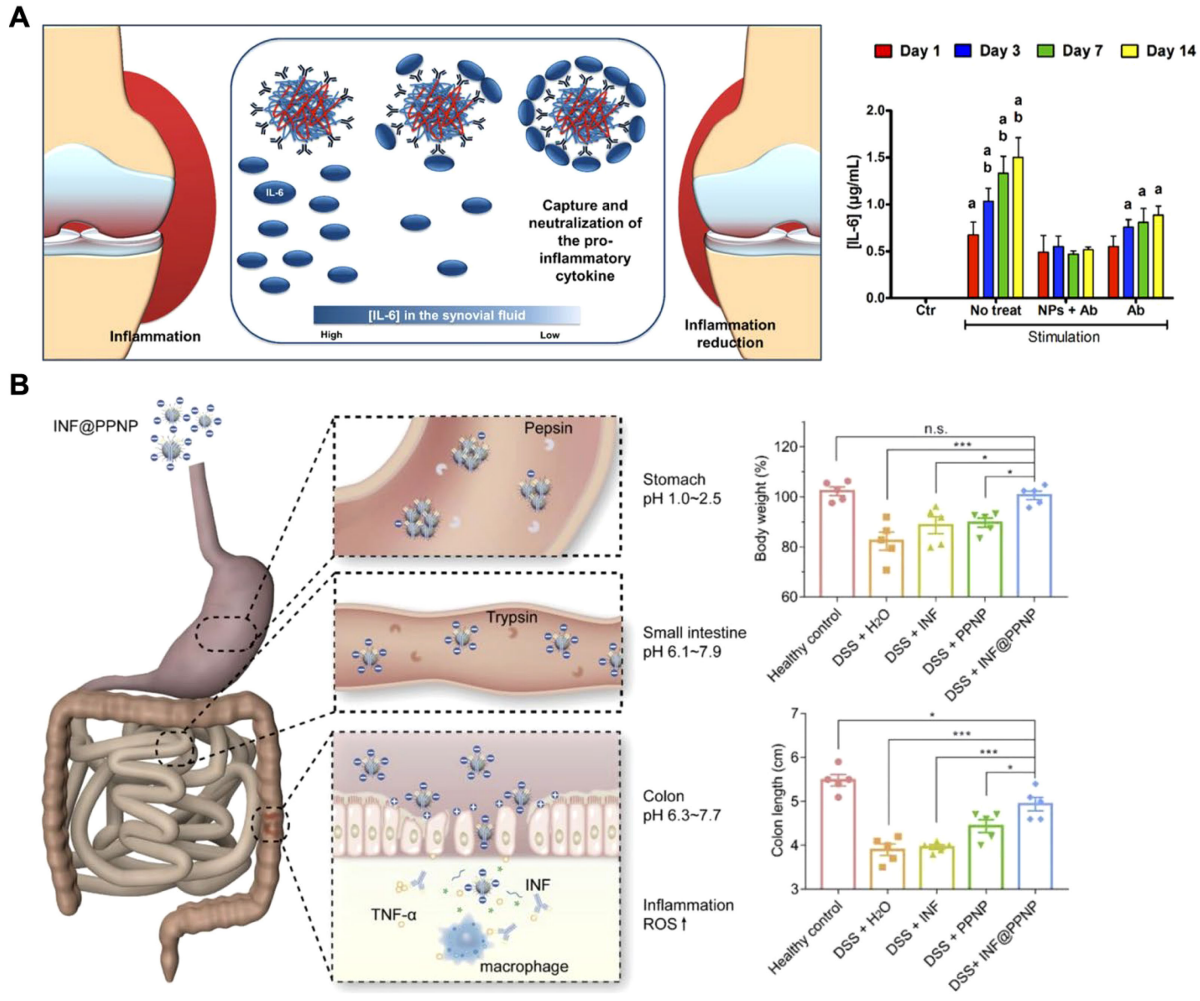
Delivery of anti-cytokine antibodies using nanoparticles. **(A)** Biodegradable nanoparticle-antibody complexes exhibit effective neutralization of IL-6 and more prolonged anti-inflammatory action compared to free IL-6 antibodies. Adapted with permission (147). Copyright 2018, American Chemical Society. **(B)** Inflammation-targeting polyphenol-poloxamer nanoparticles were developed for oral administration to treat colitis, providing gastric protection for anti-TNF antibodies and ensuring targeted release in inflamed colon tissue. Adapted with permission (153). Copyright 2020, Ivyspring International Publisher. *p < 0.05, ***p < 0.001, and n.s. = not significant.

Attaching antibodies onto nanoparticle surfaces can improve accumulation at inflammation sites, enhancing their cytokine neutralization ability (145, 146). Nanoparticle–antibody complexes offer advantages over free antibodies, including greater stability, targeted delivery, and extended retention at target sites (64). For example, researchers have developed biodegradable nanoparticles to neutralize IL-6 in arthritic diseases, demonstrating a promising approach to combating inflammation (**Figure 7A**) (147). These nanoparticles were fabricated using two naturally derived polymers, chitosan and hyaluronic acid, with anti-IL-6 antibodies immobilized on their surfaces. The surface-anchored antibodies effectively captured and neutralized IL-6, achieving approximately 98% efficiency with an initial antibody concentration of 10 μg/mL. Biological assays confirmed the cytocompatibility of this nanoplatform with human articular chondrocytes and macrophages. In co-culture systems with inflammatory M1 macrophages, the nanoparticles significantly reduced inflammatory effects on human chondrocytes and exhibited a more prolonged anti-inflammatory action compared to free IL-6 antibodies. Similar strategies have been applied to conjugate other antibodies and treat various inflammatory conditions, including inflammatory bowel disease, highlighting their potential to enhance the efficacy of existing antibody-based therapies (148, 149).

#### 3.1.2 Nanoparticle encapsulation

While the surface modification of nanoparticles with antibodies has demonstrated promise for cytokine neutralization, this approach may not be suitable for certain scenarios. In inflammatory bowel disease (IBD), for example, anti-TNF therapies are often administered via injection or intravenous delivery, which can lead to systemic side effects like increased infection risk (150). Although oral delivery could reduce these side effects, both free and nanoparticle surface-attached antibodies risk degradation in the stomach before reaching the site of inflammation (151, 152). To address this, researchers developed an oral delivery system using supramolecular nanoparticles composed of tannic acid and a lipid-PEG that encapsulated infliximab, protecting the antibody from the gastrointestinal environment (**Figure 7B**) (153). These pH-sensitive nanoparticles remained stable in the acidic stomach environment and released the antibody in the neutral or slightly alkaline conditions of the inflamed intestine. This targeted release of the antibody promoted precise anti-TNFα therapy for IBD. *In vivo* studies in a mouse colitis model demonstrated that the orally administered formulation accumulated well in inflamed colon tissue, minimizing systemic exposure and concentrating therapeutic action at the disease site. Similarly, novel biodegradable nanoparticles composed of polyesterurethane and its PEGylated derivatives, due to their enhanced cellular interaction and translocation across inflamed epithelial cells, have been utilized as nanocarriers for delivering anti-TNFα antibodies to treat epithelial inflammation (154).

### 3.2 CNPs

Nanoparticle–antibody complexes have shown potential by leveraging the advantageous properties of nanoparticles such as improved biodistribution and targeting functionality (155). However, their impact is often constrained by a focus on a limited set of cytokines, whereas inflammatory conditions are inherently complex in nature (156, 157). To overcome these limitations, researchers are turning to CNP-based nanosponges, an innovative platform that uses natural cell receptors to neutralize a wider range of cytokines (158, 159). CNPs consist of synthetic nanoparticle cores coated with natural cell membranes, which enable them to mimic cellular behavior based on the membrane type used (160, 161). This design allows CNPs to harness the natural cytokine-capturing abilities of cells via diverse receptor interactions, making them a versatile platform for the simultaneous neutralization of multiple cytokines in inflammatory conditions (64, 138).

#### 3.2.1 Macrophage membrane-coated nanoparticles

Macrophages, in particular, play a critical role in modulating inflammation and clearing cytokines due to their broad array of cytokine receptors, including those for TNFα, IL-6, and IL-1 (5, 162, 163). As a result of their simultaneous interactions with multiple inflammatory cytokines, macrophage membranes are an ideal material for multiplex cytokine neutralization (152, 164–170). Initially applied for sepsis treatment, MΦ-NPs were developed to act as decoys to capture excessive cytokines and reduce cytokine storms (170). Since then, their applications have expanded to a range of inflammatory diseases, including SARS-CoV-2 infection, rheumatoid arthritis, atherosclerosis, bone infections, and hyperuricemia.

**Figure 8:**
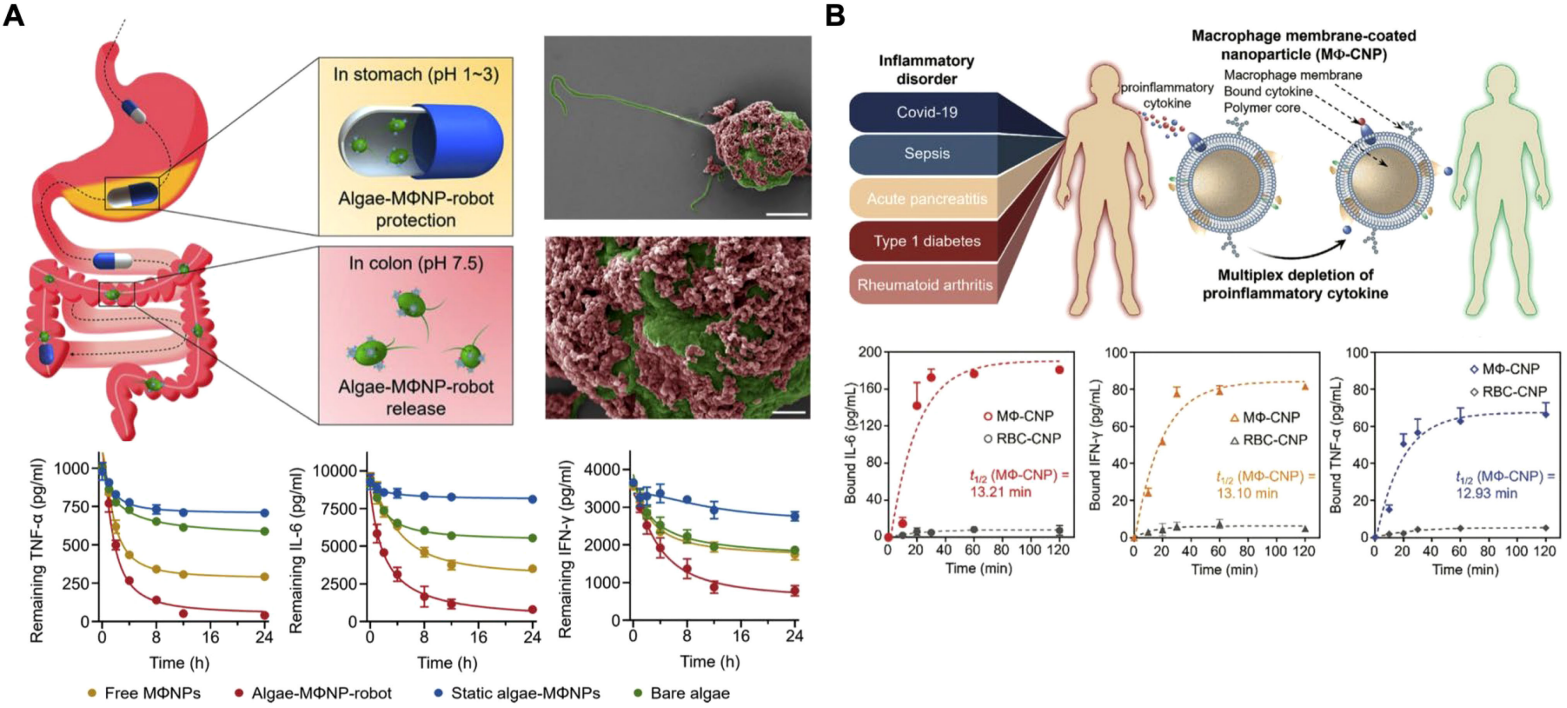
Macrophage membrane-coated nanoparticles (MΦ-NPs) for cytokine neutralization. **(A)** Motile green microalgae functionalized with MΦ-NPs are used for the active capture of proinflammatory cytokines within inflamed tissues in the gastrointestinal tract. Adapted with permission (171). Copyright 2024, American Association for the Advancement of Science. **(B)** MΦ-NPs are capable of neutralizing cytokines in human serum and synovial fluid samples from patients with inflammatory diseases. Adapted with permission (172). Copyright 2024, Wiley-VCH.

A very recent approach combined motile green microalgae with MΦ-NPs to create biohybrid microrobots for actively capturing proinflammatory cytokines (**Figure 8A**) (171). This approach integrated the active motion of cell-based microrobots with the cytokine-neutralizing capacity of macrophage membranes, enabling efficient cytokine capture without directly suppressing immune function. In studies on inflamed colitis tissue, the biohybrid robots demonstrated efficient movement and neutralization of key proinflammatory cytokines, such as TNFα, IL-6, IL-1β, and IFNγ. In a mouse model of IBD, oral administration of the platform significantly improved both prevention and treatment outcomes, demonstrating a favorable safety profile and potential for managing inflammatory diseases of the gastrointestinal tract.

Beyond animal models, MΦ-NPs have shown promise in human cytokine neutralization. In one study, researchers evaluated MΦ-NPs in human serum samples from patients with COVID-19, sepsis, acute pancreatitis, and type-1 diabetes, as well as synovial fluid from patients with rheumatoid arthritis (**Figure 8B**) (172). MΦ-NPs effectively reduced levels of IL-6, IL-8, IFNγ, and TNFα, whereas control red blood cell membrane-coated nanoparticles had minimal effect. These results demonstrated the versatility of MΦ-NPs for cytokine neutralization in various inflammatory conditions, highlighting their potential for clinical use.

#### 3.2.2 Neutrophil membrane-coated nanoparticles

**Figure 9:**
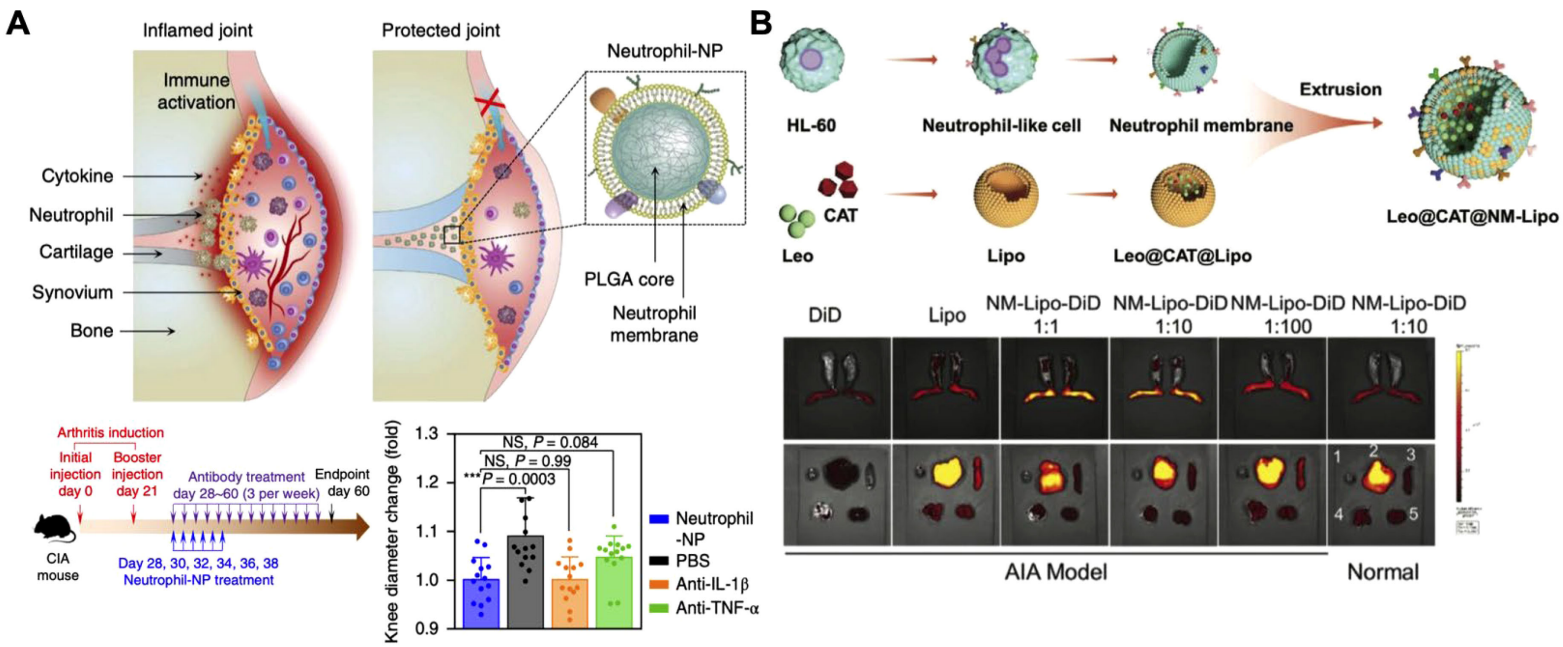
Neutrophil membrane-coated nanoparticles (Neu-NPs) for cytokine neutralization. **(A)** Neu-NPs neutralize proinflammatory cytokines in rheumatoid arthritis models. Adapted with permission (181). Copyright 2018, Nature Publishing Group. **(B)** Reactive oxygen species-responsive and oxygen-generating Neu-NPs enable the targeted treatment of refractory rheumatoid arthritis. Adapted with permission (182). Copyright 2024, Wiley-VCH.

In addition to macrophages, other immune cells, such as neutrophils, offer distinct benefits for cytokine neutralization (173–175). As one of the first responders to inflammation, neutrophils play a key role in regulating inflammatory cascades and promoting tissue repair, making Neu-NPs a valuable option for cytokine capture (176–180). For instance, in rheumatoid arthritis models, Neu-NPs selectively targeted inflamed cells, neutralized proinflammatory cytokines, reduced synovial inflammation, penetrated cartilage, and protected joints from damage (**Figure 9A**) (181). In both collagen-induced arthritis mouse models and human transgenic arthritis models, Neu-NPs demonstrated notable therapeutic benefits, reducing joint damage and arthritis severity. In another related approach, researchers developed a neutrophil membrane-coated nanoliposomal system designed for targeted rheumatoid arthritis therapy (**Figure 9B**) (182). Leonurine, a natural anti-arthritic agent, was loaded along with catalase within liposomes and subsequently cloaked with a neutrophil membrane. This formulation functioned as neutrophil-mimicking nanodecoys that targeted inflamed synovial tissues, where they absorbed inflammatory cytokines and chemokines that would otherwise have activated immune cells and amplified inflammatory responses. Additionally, the coloaded catalase facilitated oxygen production in high reactive oxygen species environments, helping to alleviate oxidative stress and hypoxia while drug was released from the nanostructure in a controlled manner. Both *in vitro* and *in vivo* assessments confirmed the formulation’s strong biosafety profile, positioning it as a promising strategy for managing various inflammation-driven diseases.

#### 3.2.3 Genetically engineered cell membrane-coated nanoparticles

**Figure 10:**
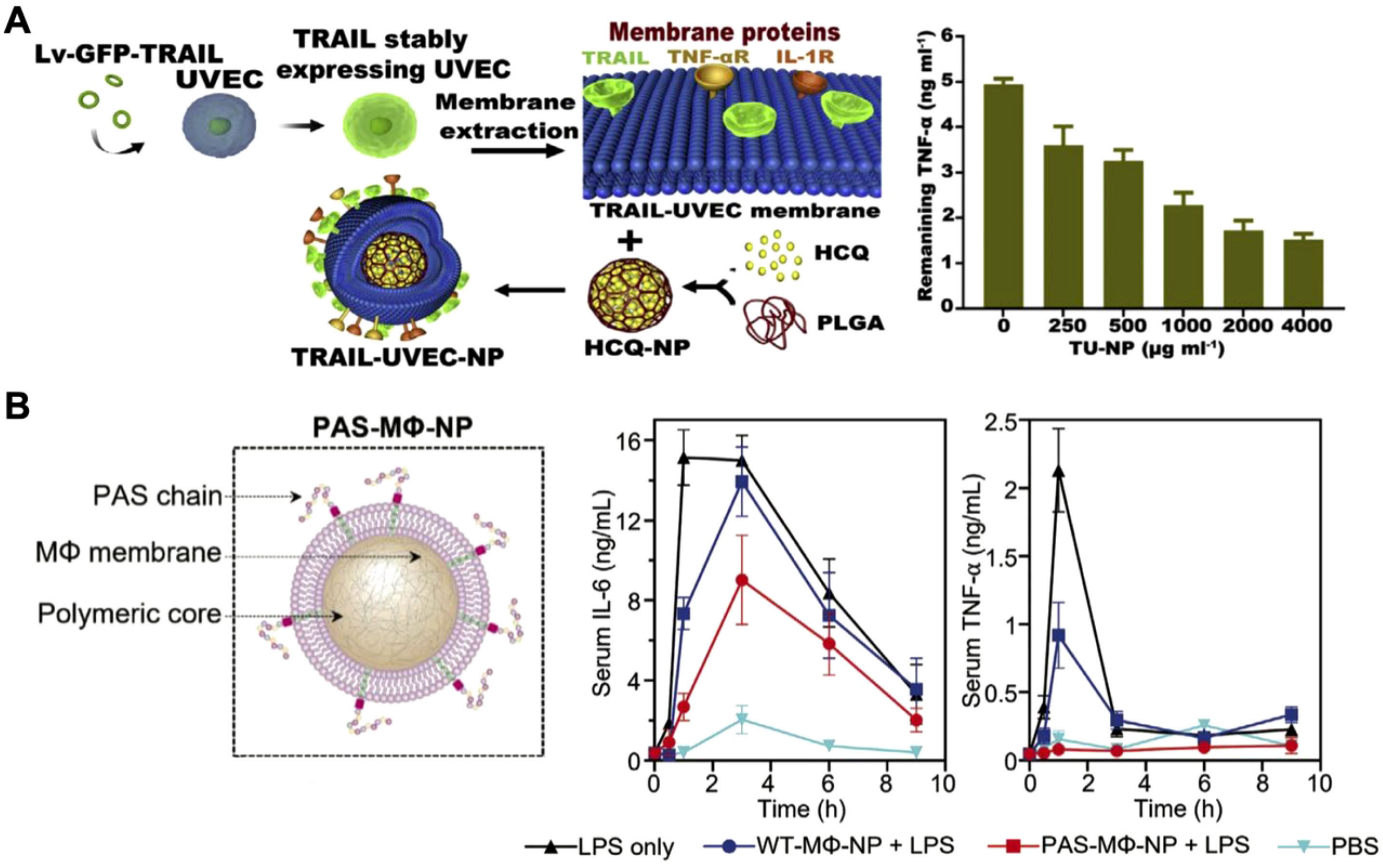
Genetically engineered cell membrane-coated nanoparticles for cytokine neutralization. **(A)** Drug-loaded nanoparticles coated with genetically engineered umbilical vein endothelial cell membranes and modified with an inflammation-targeting ligand experience enhanced accumulation in inflamed joints for effective cytokine neutralization in rheumatoid arthritis. Adapted with permission (189). Copyright 2020, Elsevier. **(B)** Macrophages genetically modified to express proline-alanine-serine (PAS) peptide chains show extended *in vivo* retention time and enhanced efficacy in suppressing inflammatory cytokines in models of lipopolysaccharide-induced lung injury and endotoxemia. Adapted with permission (190). Copyright 2023, Wiley-VCH.

While immune cell membrane-based CNPs like MΦ-NPs and Neu-NPs have demonstrated broad-spectrum cytokine neutralization capabilities, they largely depend on the natural receptor profiles and functions of the original immune cells. Challenges such as limited targeting specificity and short retention times, especially in complex disease environments, remain (137, 183). Genetic engineering offers a promising solution by allowing for customized membrane modifications that enhance targeting and extend circulation time (184–188). For example, researchers introduced the inflammation-targeting TNF-related apoptosis-inducing ligand onto umbilical vein endothelial cell membranes, which naturally express multiple cytokine receptors, and coated these membranes onto drug-loaded cores for rheumatoid arthritis therapy (**Figure 10A**) (189). In arthritis models, the nanoparticles accumulated and persisted longer in inflamed joints, neutralizing cytokines, reducing inflammation, and protecting cartilage from damage. In another study, macrophages were genetically modified to express proline-alanine-serine (PAS) peptide chains, extending the MΦ-NPs’ *in vivo* retention time (**Figure 10B**) (190). These PAS-modified nanoparticles resisted premature clearance from the bloodstream by preventing immune cell uptake. Pharmacokinetic studies showed that the modified MΦ-NPs had higher blood retention and lower liver uptake, resulting in enhanced effectiveness for suppressing inflammatory cytokines in models of lipopolysaccharide-induced lung injury and endotoxemia. This work highlights the potential of engineered cell membranes to improve targeting, retention, and overall therapeutic outcomes in cytokine neutralization.

## 4 Clinical translation

As researchers continue to innovate on nanoparticle-based cytokine manipulation strategies, a key focus now lies in translating promising technologies to the clinic. Since the initial exploration of IL-12 for cancer treatment in the 1990s, many of the earlier limitations related to dose-dependent toxicity and systemic inflammation have been addressed (41, 191). Recombinant cytokines and gene-based delivery methods have broadened the therapeutic landscape by enabling controlled, sustained cytokine expression with fewer off-target effects (35). Current clinical trials are testing various recombinant and fusion cytokines for conditions ranging from advanced solid tumors to autoimmune diseases (24, 192). For example, a recombinant fusion of IL-12 with an albumin-binding domain utilizes human serum albumin transport pathways to improve tumor accumulation and is now in a Phase I/II trial (NCT05756907) for patients with advanced solid tumors and platinum-resistant ovarian cancer (193). Similarly, M9241 (NHS-IL12), a fusion of IL-12 heterodimers with the NHS76 antibody, targets necrotic tumor regions for precise cytokine release within the tumor microenvironment and is being tested in combination with chemotherapy for metastatic colorectal cancer in a Phase II trial (NCT05286814) (194). In terms of autoimmune disease treatments, efavaleukin alfa, a PEGylated IL-2 developed by Amgen that selectively expands regulatory T cells and exhibits prolonged circulation, is currently in a Phase I trial (NCT04987333) for systemic lupus erythematosus and chronic graft-versus-host disease (195).

Gene delivery methods, including plasmid and mRNA platforms, have expanded cytokine therapy options by enabling sustained expression with reduced systemic side effects (42, 196). EGEN-001, an IL-12-encoding plasmid formulated with PEG-PEI-cholesterol lipopolymers to enhance cellular uptake, is in a Phase II trial (NCT01118052) for persistent or recurrent epithelial ovarian cancer (197). Another example, NNC0361-0041, a Novo Nordisk-developed plasmid encoding pre-proinsulin, IL-2, IL-10, and TGF-β1, is now in a Phase I trial (NCT04279613) for type 1 diabetes, highlighting the versatility of gene therapies (198). The COVID-19 pandemic has further catalyzed the development of mRNA-based cytokine therapies, with LNPs emerging as effective and safe delivery vehicles. For example, Moderna’s mRNA-6231, encoding IL-2 fused with human serum albumin, has completed a Phase I trial (NCT04916431) to evaluate its safety and efficacy for potential autoimmune applications (199). Similarly, BioNTech’s mRNA-based BNT153 and BNT152, encoding IL-2 and IL-7, respectively, are in a Phase I trial (NCT04710043) for advanced solid tumors (200).

In parallel with cytokine delivery, being able to manage excessive or dysregulated cytokine activity is also important, particularly in conditions like COVID-19 and sepsis where cytokine storms can lead to severe immune imbalance (201, 202). Antibody-based therapies remain a cornerstone of cytokine neutralization (49, 203). For instance, tocilizumab, an IL-6 receptor antagonist, has shown efficacy when applied to COVID-19-related cytokine storms (NCT04306705) (204) and rheumatoid arthritis (NCT06475508) (205). Adalimumab, a TNFα inhibitor, is similarly under investigation for autoimmune conditions such as rheumatoid arthritis (NCT03938701) (206) and Crohn’s disease (NCT01564823) (207). Alongside these, nanoparticle-based solutions are being explored as targeted alternatives for cytokine modulation. Mesenchymal stem cell-derived exosomes, for example, influence inflammation by adjusting anti-inflammatory cytokine levels and promoting regeneration, and they are currently being evaluated for COVID-19-related pneumonia when delivered by inhalation in a Phase I/II trial (NCT04602442) (208). These developing approaches aim to deliver safer, more targeted options for managing cytokine dysregulation, offering promise across a range of inflammatory and autoimmune conditions.

## 5 Summary and outlook

Cytokines are an essential component of the immune system, and they can be manipulated to achieve therapeutic outcomes in cancer, infectious diseases, and autoimmune disorders (1, 2). While advances in immunocytokines and antibody-based therapies have shown promise, challenges like dose-limiting toxicity remain (209, 210). In this review, we highlighted key cytokines involved in various disease contexts, followed by an exploration of their delivery in various formats—from proteins to DNA and mRNA. Emerging nanoparticle technologies can enhance cytokine targeting, stability, and controlled release. In addition, compared to traditional antibody-based cytokine neutralization methods, nanoparticle strategies offer advantages in terms of precision and multiplexing. In particular, technologies like CNPs have shown substantial promise for broad-spectrum cytokine neutralization, expanding possibilities for the effective treatment of immune-related diseases.

Many of the recent clinical trials have focused on the delivery of cytokine-encoding mRNA using LNPs, underscoring the promise of this approach. Looking ahead, further clinical testing of nanoparticle-based cytokine therapies is anticipated, supported by encouraging results in recent preclinical studies. Improved understanding of nanoparticle structure–function relationships will assist in further optimizing pharmacokinetics and targeting precision (58, 60, 211). Advanced design concepts, such as multifunctionality and stimuli-responsiveness, or assistance from machine learning, may further enhance control and minimize systemic side effects (212, 213). Importantly, long-term efficacy and safety in the context of complex diseases will be essential for regulatory approval and clinical translation of nanoparticle-based therapies (214, 215). For CNP-based cytokine nanosponges, critical considerations will include the choice of cell membrane sources, cross-species reactivity, and regulatory challenges. Also, combining cytokine-manipulating nanoparticles with established therapies, including immune checkpoint inhibitors and chimeric antigen receptor T cell therapy, may improve therapeutic outcomes (216–218). Integration with diagnostic tools will also aid in identifying patients who will benefit most, supporting personalized nanomedicine approaches (219, 220). Overall, nanoparticle-based cytokine delivery and neutralization strategies offer significant potential for advancing the treatment of immune-mediated diseases.
